# Heritabilities of Directional Asymmetry in the Fore- and Hindlimbs of Rabbit Fetuses

**DOI:** 10.1371/journal.pone.0076358

**Published:** 2013-10-10

**Authors:** Matteo Breno, Jessica Bots, Stefan Van Dongen

**Affiliations:** Evolutionary Biology Group, Department of Biology, University of Antwerp, Antwerp, Belgium; San Francisco Coordinating Center, United States of America

## Abstract

Directional asymmetry (DA), where at the population level symmetry differs from zero, has been reported in a wide range of traits and taxa, even for traits in which symmetry is expected to be the target of selection such as limbs or wings. In invertebrates, DA has been suggested to be non-adaptive. In vertebrates, there has been a wealth of research linking morphological asymmetry to behavioural lateralisation. On the other hand, the prenatal expression of DA and evidences for quantitative genetic variation for asymmetry may suggest it is not solely induced by differences in mechanic loading between sides. We estimate quantitative genetic variation of fetal limb asymmetry in a large dataset of rabbits. Our results showed a low but highly significant level of DA that is partially under genetic control for all traits, with forelimbs displaying higher levels of asymmetry. Genetic correlations were positive within limbs, but negative across bones of fore and hind limbs. Environmental correlations were positive for all, but smaller across fore and hind limbs. We discuss our results in light of the existence and maintenance of DA in locomotory traits.

## Introduction

The causes of phenotypic variation in morphology and behaviour, key factors of adaptability and survival, remain poorly understood in some areas. A case in point is represented by morphological and behavioural lateralization (e.g., [1]). Although the external metazoan body plan is assumed to be symmetric, many cases of consistent differences between a pair of morphological structures have been reported (called directional asymmetry, abbreviated as DA; e.g., [2]). Some are very conspicuous, such as the morphology of flounders, spiraled snails or the ears of owls. More often, very subtle instances of DA have been observed, such as wings in insects, mandibles in mice or arms and legs in humans [3]–[5]. The evolutionary importance of these asymmetries has been the subject of many debates and appears to be unresolved. In a recent review of DA in insect wing size, Pélabon and Hansen [5] concluded that the small magnitude and imprecise expression of DA in insect wings precludes it from playing a major adaptive role. In addition, in spite of relatively few studies, the heritability of DA in insect wing size (studied in *Drosophila melanogaster*) appears very low at best ([2]; Table 1). In contrast, in vertebrates, where the genetic basis of DA has been mainly studied in humans and in mice mandibles, moderate to high levels of genetic variation have been demonstrated (Table 1). Directional asymmetries in extremities of vertebrates have been suggested to be of environmental origin and invoked to demonstrate behavioural lateralization [6]. In humans with extreme lateralization of behaviour, handedness as a cause of morphological asymmetries has been suggested repeatedly and studied intensively for over a century [7]–[9]. Because skeletal elements undergo remodelling during development [4], [10], asymmetrical loading of limbs likely causes morphological asymmetries [4], [11]–[13]. Furthermore, in humans, directional asymmetry appears to increase with age, possibly due to sustained mechanical loading [14]. DA of the upper extremities also increases with years of heavy working [15], it appears larger in upper extremities [16] and handedness is correlated with hand asymmetry [17]. All these studies emphasized the importance of environmentally determined DA. In humans, the upper limbs often show a right biased asymmetry –consistent with the prevalence of right handed individuals over left handed – whereas often, but to a lesser extent, the legs show DA in the opposite direction. This is thought to be a reflection of a compensatory action of legs in right-handed individuals. This so-called cross-asymmetry is also observed at the individual level through negative correlations in asymmetry between bones of the upper and lower extremities [18]. On the other hand, some have found directional asymmetry in fetal limbs free of mechanic loading (although lateralization in movements also occur in human fetuses; [19]) suggesting a pre-adaptation to handedness during adult life (but results are mixed; see [18]). Although humans have been studied most extensively, correlations between DA and lateralization appear to occur in many vertebrates (e.g., [6], [20]). Nevertheless, most studies cannot reveal a causal link between morphological asymmetry and behavioral lateralization [20]. Indeed, Kraak [21] suggested that DA may be a by-product of the asymmetric development of the internal body plan, of which signs emerge during the very first stages of embryological development.

**Table 1:** Overview of estimates of genetic variation in directional asymmetry in the scientific literature.

Species	Traits	Analysis^a^	h^2^	Reference
*Drosophila* *melanogaster*	number ofbristles	SEL	3.60%	[41]
*Drosophila* *melanogaster*	wingfolding	SEL	−5.80%	[42]
*Drosophila* *melanogaster*	eye size	SEL	0	[43]
*Drosophila* *melanogaster*	number ofbristles	SEL	−0.049%	[44]
*Mus musculus*	mandible	QTL	4.40%	[45]
*Arabian horse*	legmarkings	SEL	1–2%	[46]
*Homo sapiens*	dentalarch	SIB	0–33%	[47]
*Mus musculus*	mandible	PO	21%	[3]
*Drosophila* *melanogaster*	wing	SIB	>0	[48]
*Homo sapiens*	dermatoglyphics	PO/SIB	8–24%	[49]
*Homo sapiens*	ears andextremities	PO/SIB	10–20%	[50]
*Homo sapiens*	Incisoremergence	TWIN	71–96%	[51]
*Homo sapiens*	digitratio	SIB	6%	[52]
*Drosophila* *melanogaster*	cross-vein	SEL	0%	[2]
*Vulpes vulpes*	skeletaltraits	AM	N.S.	[40]

a SEL: selection experiment; QTL: Quantitative Trait Locus analysis; PO: Parent offspring regression; SIB: sib-analysis; TWIN: twin resemblance; AM animal model.

Although lateralization is often observed and studied at the population level, where on the average morphology and behaviour are biased in a particular direction, the degree of lateralization may differ among individuals. This observation leaves us with the intriguing question of what factors maintain this variation. In humans, left handedness appears to be maintained at a low frequency (≈10%) throughout its evolutionary history [22]. The lower survival and increased health problems of left-handed individuals have been suggested to be balanced by some benefits like unpredictability in fighting [23]. In reptiles, DA appears to be related to injuries and possible predator avoidance abilities [20], [24]. In this sense, DA and behavioral lateralization can be viewed as a quantitative trait that is able to respond to selection. Overall, the relative importance of a genetic predisposition to develop asymmetrically versus the effects of differences in mechanic loading between sides remains largely unknown. A crucial part of information that is lacking is to what extent variation in DA is genetically determined and emerges already during embryological development before remodelling due to mechanic loading occurs. In this perspective we study between-family variation in DA of long bones of rabbit fetuses. More specifically we investigate the following questions: i) does DA exist in the long bones of rabbit fetuses and how accurately is it expressed?; ii) is DA higher in bones of fore limbs?; iii) is there evidence for cross-symmetry at either the population and/or individual level?; and iv) is there evidence for genetic variation and/or genetic correlations in DA?

## Materials and Methods

We obtained data from two toxicological experiments aiming at assessing the effect of two compounds (of which we cannot disclose the names due to company policy) on embryo-fetal development. Both compounds were administered orally by gavage to pregnant New Zealand White rabbits and their developing offspring *in utero* once daily during the period of organogenesis (from Day 6 to 19 inclusive of pregnancy). Pregnant females originated from a large outbreed population. Each experiment is composed of three test article dosed groups (100, 500 and 1500 mg/kg for the first experiment with compound A and 80, 320, 1280 mg/kg for the second one with compound B) and one vehicle group (0 mg/kg) for both experiments. Clinical signs of toxicity, body weight performance and food consumption were recorded. The females were killed on Day 28 of pregnancy (gestational length is about 30 days in this species) and a necropsy was performed during which the females were examined for macroscopic abnormalities, pregnancy status, the numbers of corpora lutea of pregnancy, implantations, early and late resorptions and live and dead fetuses. The fetuses were weighed and examined for external, visceral and skeletal abnormalities. All fetuses were processed for skeletal examination by staining of the bones with Alizarin red. Compound A was an anti-hyperglycemic agent for the treatment of adults with diabetes type II. Its development has been stopped because of the lack of therapeutic effects in clinical trials and the compound as such is no longer produced. Compound B is used for the prevention and treatment of coccidiosis in broiler chickens and growing turkeys. This anti-protozoal agent for veterinary use works locally in the gastrointestinal tract and has very limited systemic exposure. Compound A resulted in maternal toxicity in the groups receiving 500 and 1500 mg/kg as evidenced by a decrease in body weight and food consumption. Related to the maternal toxicity lower fetal weights were recorded at term, as well as incomplete ossification of several bones at skeletal examination, but no other developmental abnormalities related to the treatment were observed. Compound B directly affected the development of the foetuses with an increase in number of malformed fetuses in the high dose group receiving 1280 mg/kg [25]. The most common abnormalities involved nasal and frontal bones for the skull, reduced ossification of axial elements, the presence of a rudimentary or complete 13th rib, fused or rudimentary sternum, reduced ossification of the pubis [25]. None of the compounds directly affected limb development [25], except for a reduced ossification of the tarsal bone is few cases (≈3%). This work had been approved by the ethical committee for animal experimentation of Janssen Pharmaceutica N.V.

**Figure 1:**
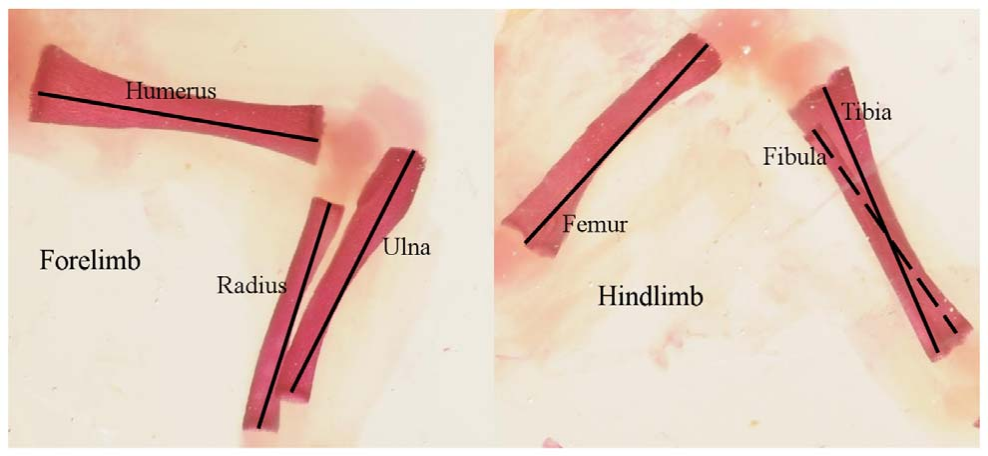
Six long bones (humerus, radius, ulna, femur, tibia, fibula) measured on pictures of rabbit fetuses. The skeleton is stained with Alizarin red.

The joined number of fetuses sum up to 1126 divided over 133 litters (with a median litter size of 9, ranging from 1 to 14). Six traits were studied: humerus, ulna, radius, femur, tibia and fibula (Figure 1). Each trait was photographed twice after independent repositioning of the fetus. Each picture was measured once or twice (repeated measures of the same picture were taken for approximately 40% of the dataset). Two operators (MB and JB) measured half of each dataset, and a preliminary analysis showed no difference due to operator handling. Since it was impossible to measure all traits in all individuals due to damaged parts, the number of individuals measured ranged from 1018 for humerus to 1097 for radius. For each trait and each experiment we ran a mixed regression model to obtain unbiased individual asymmetry values [26], i.e., the signed FA. Measurement error ranged between 30 and 50% and was mainly due to the positioning of the foetuses. Correlations in the signed FA between the two operators, computed on a subset of the dataset measured by both, ranged between 0.54 and 0.82.

Analyses were based on mixed models with the signed FA as response variable. Treatment, experiment, and their interaction were added as fixed effects to correct for possible confounding effects of the applied compounds when merging data from the two experiments. Family, nested within treatment and experiment, was added as random effect. We applied a Bayesian model with weak priors and MCMC sampling to obtain the posterior distributions of the model parameters. The heritability was estimated as h^2^ = σ^2^_dam_/(σ^2^_dam_+σ^2^_res_), and the coefficient of between family variance as CVB = σ_dam_/µ_DA_. Posterior distributions were summarised as the mean, standard error and 95% highest posterior density (HPD interval). The same model was applied to the multivariate dataset with residuals and family effect correlated in order to compute genetic and environmental correlations among bones. Phenotypic correlations at the individual level were tested using Pearson’s correlation coefficients. To test whether forelimbs displayed higher levels of DA, a pairwise comparison between elements belonging to different limbs was computed by means of an ANCOVA (adding trait size and experiment as covariate), changing one trait’ sign when necessary; a Bonferroni correction for multiple testing was applied.

In order to explore if the data from the two experiments could be analysed in a single analysis (mainly to minimize standard errors of the genetic parameter estimates), we compared levels of DA and tested for treatment effects. In addition, estimates of genetic parameters were obtained for each experiment separately as well. For each trait, a mixed model was run with family as random effect and treatment nested within experiment as fixed effects. Finally, as specific maternal reaction to the treatment may inflate between family variance, and our study design did not allow to explicitly test for such effects, we performed sensitivity analyses. First, we estimated the genetic parameters for the control groups only (i.e., untreated dams only). In addition, if between-family variation in DA would be increased by between-dam variation in the effects of treatment, w hypothesis that this would be expressed most strongly in the high dose group (see above; the dose group showing significant treatment effects on development). We therefore excluded this dose group only and compared results to the genetic parameters obtained for the entire experiment.

All analyses were performed in R (version 2.10) [27] and the Bayesian analysis was performed using the MCMCglmm (Hadfield 2010) [28] package for R.

## Results

### Effects of Treatment on Directional Asymmetry and Comparisons between Experiments

Likelihood ratio tests for fixed effects showed that treatment did not have any effect on DA (all *P>*0.05), while differences in DA between experiments was significant for three traits only (Radius χ^2^_1_ = 10.15, *P<0.01*; Femur χ^2^_1_ = 6.8, *P<0.01*; Fibula χ^2^_1_ = 7.11, *P<0.01*; Humerus: χ^2^_1_ = 0.29, *P>0.10*; Ulna: χ^2^_1_ = 2.94, *P>0.10*: Tibia: χ^2^_1_ = 2.05, *P>0.10* ). Average DA across treatments in both experiments were reported in Table 2 and did not show any consistent differences.

**Table 2:** Descripive statistics (mean and standard error) of directional asymmetries (cm ×10^−3^) in the different traits for each dose level in the two experiments.

Experiment 1					
Trait	Control	Low	Medium	High	?^2^_3_
Humerus	1.58 (0.4)	2.01 (0.4)	1.69 (0.4)	1.11 (0.4)	2.42
Ulna	−1.13 (0.7)	−0.24 (0.7)	−0.82 (0.7)	−2.07 (0.7)	3.61
Radius	1.41 (0.3)	1.77 (0.3)	1 (0.3)	1.54 (0.3)	0.94
Femur	−0.19 (0.3)	−0.26 (0.3)	−0.12 (0.3)	0.2 (0.4)	0.95
Tibia	−1.13 (0.5)	−0.04 (0.5)	−0.51 (0.5)	−1.26 (0.5)	4.59
Fibula	−0.81 (0.3)	−0.34 (0.3)	−0.84 (0.3)	−0.36 (0.3)	2.92
**Experiment 2**					
**Trait**	**Control**	**low**	**medium**	**high**	**χ^2^_3_**
Humerus	2.79 (0.7)	2.6 (0.8)	0.97 (0.8)	0.52 (0.8)	6.28
Ulna	−0.99 (0.7)	−1.97 (0.8)	−2.35 (0.8)	−2.76 (0.9)	2.86
Radius	0.6 (0.4)	0.72 (0.4)	0.4 (0.4)	0.67 (0.5)	0.34
Femur	−1.12 (0.6)	−1.15 (0.6)	−0.24 (0.7)	−1.26 (0.7)	1.66
Tibia	−0.35 (0.4)	−0.4 (0.5)	−0.6 (0.5)	0.47 (0.5)	2.83
Fibula	0.11 (0.5)	0.04 (0.5)	0.39 (0.6)	0.19 (0.6)	0.25

Results of the likelihood ratio test for treatment−effects are also added.

Estimates of the genetic parameters are shown in Table 3. Average DA was generally consistent across experiment, except for those traits that displayed an average DA not different from zero (Table 3). Coefficients of variation were very high suggesting low developmental precision. Heritabilities were generally higher for experiment 2 (except for tibia), but with confidence intervals largely overlapping between the two experiments. Genetic and environmental correlations were similar to those for the joined dataset (see below) in their point estimates, but with broader confidence intervals often encompassing zero (details not shown). Whether the differences between the two experiments are the effect of sampling variation (due to the reduction of sample size) or are somewhat real remains uncertain.

**Table 3:** Descriptive statistics of the degree of directional asymmetry (right-left) and estimates of the heritability and coefficient of between family variance (CVB) for the two experiments separately and for the combined dataset.

Trait	Directional asymmetry (cm ×10^−3^)				Heritability and coefficient of between familyvariance of directional asymmetry			
	Experiment 1							
	Mean (SE)	95% CI	CV %	%	h^2^ (SE)	95% CI	CVB (SE)	95% CI
Humerus	**1.61** (0.19)	1.23–1.96	275.02	0.17	0.09 (0.05)	0–0.18	56 (19)	10–88
Ulna	−**1.01** (0.34)	−1.68–−0.33	711.03	0.11	0.12 (0.06)	0–0.24	171 (47)	86–269
Radius	**1.43** (0.15)	1.12–1.7	243.87	0.18	0.07 (0.05)	0–0.16	44 (17)	3–73
Femur	−0.13 (0.16)	−0.46–0.18	3028.4	0.01	0.12 (0.06)	0–0.22	710 (197)	315–1093
Tibia	−**0.72** (0.21)	−1.14–−0.3	702.19	0.07	0.11 (0.06)	0–20	155 (48)	63–251
Fibula	−**0.59** (0.15)	−0.88–−0.31	590.99	0.06	0.06 (0.05)	0–16	96 (45)	1–167
	**Experiment 2**							
	**Mean (SE)**	**95% CI**	**CV %**	**%**	**h^2^ (SE)**	**95% CI**	**CVB (SE)**	**95% CI**
Humerus	**1.83** (0.41)	0.98–2.58	283.71	0.18	0.34 (0.12)	0.11–0.56	117 (24)	70–163
Ulna	−**1.94** (0.4)	−2.72–−1.21	291.52	0.2	0.33 (0.1)	0.14–0.53	118 (21)	78–160
Radius	**0.61** (0.2)	0.21–1.01	497.9	0.08	0.18 (0.1)	0–0.34	141 (57)	0–215
Femur	−**0.97** (0.3)	−1.56–−0.4	469.03	0.1	0.26 (0.09)	0.1–0.46	168 (35)	102–236
Tibia	−0.23 (0.23)	−0.7–0.2	1862.38	0.02	0.01 (0.02)	0–0.04	40 (89)	0–241
Fibula	0.17 (0.27)	−0.34–0.73	2342.55	0.02	0.42 (0.1)	0.22–0.62	1074 (163)	773–1413
	**Combined**							
	**Mean (SE)**	**95% CI**	**CV %**	**%**	**h^2^ (SE)**	**95% CI**	**CVB (SE)**	**95% CI**
Humerus	**1.72** (0.2)	1.33–2.08	279.46	0.17	0.18 (0.06)	0.07–0.29	82 (14)	57–112
Ulna	−**1.38** (0.26)	−1.89–−0.89	303.21	0.14	0.18 (0.05)	0.09–0.28	146 (21)	104–186
Radius	**1.1** (0.13)	0.86–1.35	483.96	0.14	0.12 (0.05)	0.03–0.22	74 (16)	42–103
Femur	−**0.45** (0.17)	−0.79–−0.11	909.05	0.05	0.19 (0.05)	0.09–0.29	284 (40)	212–375
Tibia	−**0.53** (0.16)	−0.84–−0.23	910.24	0.05	0.05 (0.05)	0.00–0.14	113 (88)	0–240
Fibula	−**0.29** (0.14)	−0.54–0	1158.92	0.03	0.21 (0.05)	0.11–0.32	376 (52)	276–475

Values in bold are statistically significant from zero at P<0.01 based on a simple t-test. Forelimb: humerus, ulna and radius; Hindlimb: femur, tibia and fibula.

Since we could not exclude that between-family variation in DA would be biased upward due to dam-specific reactions to the treatment effect, we performed two sensitivity analyses. In both cases (i.e., estimating genetic parameters for the control groups only and excluding the high dose group), however, results were comparable (though with wider confidence intervals). Thus, we did not find any indication that treatment had any effect on either average DA (see above), or the genetic parameters estimated. Thus, we anticipated that conclusions from the two experiments separately would not be drastically different from those obtained on the combined dataset, and because we do not have a priori reason to consider them different populations, the two experiments were pooled, leading to much narrower confidence intervals.

### Analysis of the Combined Dataset

**Figure 2:**
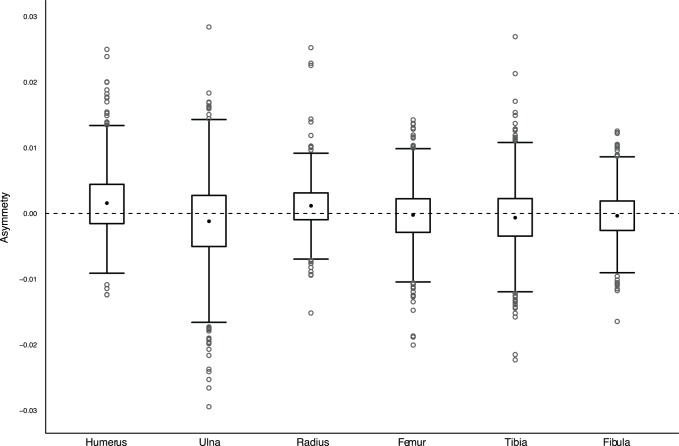
Boxplots displaying the distribution of directional asymmetry (right-left) for each trait (in cm).

Variation in DA across traits is shown in Figure 2. Each of the six traits showed evidence of directional asymmetry as zero was not contained in the 95% CI of the average asymmetry (Table 3). For humerus and radius DA was right biased, for all others it was left biased. On average, albeit only small for each bone, the degree of DA was higher for forelimbs (≈0.15% of trait size) compared to hindlimbs (≈0.05% of trait size) (Table 3). Coefficients of phenotypic variation were high, ranging from ∼300 to ∼1000%, reflecting small average DA and a very imprecise expression (Hansen et al. 2006 [29]). Pairwise comparisons of forelimb vs. hindlimb elements, corrected for multiple testing, showed that DA was higher in forelimbs (*P<0.01* for all comparisons). Marginally significant differences were observed for radius vs. femur and fibula (*P* = 0.015 and *P = *0.03 respectively), whereas no difference was observed between radius and tibia (*P* = 0.8). Correlations in the signed asymmetry were positive among traits within the fore- and within the hindlimb, but not statistically significant between bones of the fore- and the hindlimb (Table 4).

**Table 4:** Between-trait correlations in signed asymmetry.

	Humerus	Ulna	Radius	Femur	Tibia
Ulna	**0.3**				
Radius	**0.23**	**0.19**			
Femur	−0.03	0.02	−0.02		
Tibia	0.03	0.04	−0.01	**0.33**	
Fibula	0.07	−0.06	0.01	**0.19**	**0.32**

Statistically significant correlations (P<0.001) are highlighted in bold. Forelimb: humerus, ulna and radius; Hindlimb: femur, tibia and fibula.

Between-family variation comprised about 20% of the total phenotypic variation in the signed asymmetry as heritabilities ranged between 0.12 and 0.21 (Table 3). Heritabilities were estimated accurately as the lowest limit of the 95% HPD equalled 0.03, and the highest upper limit 0.32 (Table 3). Tibia was somewhat an exception with h^2^ of 0.05 and confidence intervals bounded to zero (Table 3).

CVBs were all very high, especially for the hindlimbs, which can be attributed to the very low levels of mean DA (Table 3). A multivariate mixed model was applied to standardized signed asymmetry to provide a covariance matrix at family level. These covariances were used to compute genetic correlations among traits. Table 4 shows the median and the 95% HPD intervals for genetic correlations among traits. Within limb genetic correlations were positive and the 95% HPD intervals never included zero. Their point estimates where generally higher than the phenotypic correlations (see Table 4). Genetic correlations between bones of fore and hind limbs were all negative, but the 95% HPD intervals showed that only three of them could be considered significantly higher than zero (Table 5). Environmental correlations computed from residual variances and covariances are shown in Table 5. Correlations among traits within fore and hind limbs were similar to the phenotypic ones (see Table 3) and their 95% HPD interval did not include zero. Environmental correlations in signed asymmetry between bones across fore and hind limbs were low, but significantly positive in three cases (while the phenotypic correlations were not; see Tables 4 and 5).

**Table 5:** Genetic and environmental correlations (below and the above the diagonal) and their respective 95% HPD intervals.

	Humerus	Ulna	Radius	Femur	Tibia	Fibula
Humerus		**0.29** (0.23–0.34)	**0.21** (0.15–0.27)	0.02 (−0.06–0.08)	**0.07** (0.01–0.14)	**0.11** (0.05–0.17)
Ulna	**0.4** (0.05–0.72)		**0.16** (0.09–0.22)	0.03 (−0.03–0.10)	**0.07** (0.004–0.13)	−0.01 (−0.08–0.05)
Radius	**0.45 (**0.04–0.77**)**	**0.44** (0.04–0.77)		−0.03 (−0.11–0.03)	0.02 (−0.05–0.08)	0.07 (−0.003–0.13)
Femur	−0.38 (−0.71–0.05)	−0.21 (−0.62–0.19)	−0.12 (−0.56–0.33)		**0.30** (0.23–0.36)	**0.18** (0.11–0.24)
Tibia	−**0.65** (−0.89–−0.29)	−0.31 (−0.69–0.09)	−0.18 (−0.63–0.28)	**0.55** (0.16–0.82)		**0.31** (0.26–0.37)
Fibula	−**0.42** (−0.76–−0.02)	−**0.40** (−0.76–−0.02)	−0.40 (−0.77–0.03)	**0.53** (0.17–0.80)	**0.59** (0.23–0.83)	

Statically significant results are indicated in bold. Forelimb: humerus, ulna and radius; Hindlimb: femur, tibia and fibula.

## Discussion

### Directional Asymmetry at the Phenotypic Level

Directional asymmetry has been considered a paradoxical trait [2] because despite the fact that DA evolved multiple times [30], evidence for additive genetic variation remains controversial [2], [5]. Moreover, the link between behavioural lateralization and DA and the emergence of asymmetry early in life need further elucidation. For this reason, we have investigated several hypotheses concerning DA in fetal limbs of the New Zealand white rabbit.

Magnitude of DA, expressed as percentage of the average trait size, was small compared to other studies but within the reported range, in particular for fetal traits [14], [18]. Nevertheless, all traits displayed highly significant levels of DA, in at least one of the experiments and overall, which highlights the presence of DA already at the end of fetal life. In general, forelimbs possessed higher DA than hindlimbs, and DA was similar across traits belonging to the same limb. Whether these results suggest that DA plays a functionally different role in forelimb and hindlimb remains to be determined. An increase in upper limb asymmetry compared to lower limbs has been consistently reported in human literature and interpreted as consequence of freeing upper limbs from locomotion, which in turn would constrain lower limbs toward symmetry [4]. Whether differences in DA among limbs, in rabbits, could be explained by stronger functional constrains on hindlimbs is currently unknown. In the only study we are aware of, Garland and Freeman [31] reported a decrease in DA in hindlimbs of mice selected for high endurance running, suggesting that symmetry may be adaptive. However, as Garland and Freeman [31] did not study forelimbs any functional explanation remains speculative, especially considering the high developmental imprecision of DA (see below). Alternatively, DA may arise as a by-product of early development. For example, genes involved in early establishment of a left-right embryonic axis play also a role in hindlimb development (e.g. *pitx 1* and *pitx2*; [32], [33]). Another example came from toxicological studies, where the right bias of cadmium induced limb malformations was ascribed to the asymmetric vasculature that supplies the early limb bud [34].

Phenotypic correlations in asymmetry were all positive and significant within, but weak and not significant between elements of fore- and hindlimbs. The lack of an association in signed asymmetry between traits across fore and hind limbs indicates that forelimb and hindlimb in our dataset are not developmentally integrated (i.e. they do not share developmental noise [35], but see below for environmental correlations). Early findings in rabbits [36] demonstrated limb contro-lateral average asymmetry in bone weight. Although the direction of average asymmetry in humerus and radius was opposite to that of hindlimb bones, we did not find any negative correlations between hindlimb and forelimb at the phenotypic level. This result suggests a weak or even absent expressed cross-asymmetry.

### Quantitative Genetic (co-)variance in Directional Asymmetry

Quantitative genetic analysis revealed the presence of low to moderate heritabilities for DA in all studied traits. Assuming the absence of strong maternal effects, our genetic estimates should be considered an upper limit to the real heritability since between family variation contains dominance and epistatic variance as well. Nonetheless, our findings are in line with those reported by other authors in vertebrates (Table 1) and suggest that DA of long bones in limbs is under genetic control as well. The relative importance of non-additive genetic variance remains speculative. One could argue that it may be relatively important given the very low realized heritabilities in selection experiments (Table 1). However, these selection experiments have almost solely been performed in fruit flies, suggesting that further research in other organisms is clearly needed.

The design of our study does not allow excluding genotype-by-environment interactions. Thus our heritability estimates may be biased upward if dams react differently to the treatment. However, we consider this unlikely for two reasons. First, we found no indication that the treatments affected average levels of directional asymmetry. Second, two sensitivity analyses (controls only and excluding high dose groups) did not show any marked reductions in the estimated heritabilities. We therefore conclude that our results are robust and unlikely to be substantially biased upward due to genotype-by-environment interactions.

Within fore and hind limbs, we observed a correspondence between genetic and phenotypic correlations, since all genetic correlations were positive and even somewhat higher for hindlimbs indicating a genetic integration of DA. Interestingly, we found negative genetic correlations between fore and hindlimbs, three of which were statistically significant. Negative associations in asymmetry at the phenotypic level between front and hindlimbs have been reported in humans [4] and are usually interpreted as environmentally induced cross-asymmetry, arising from a compensation of the right handedness in the upper extremities. However, Van Dongen et al. [18] found evidence for negative phenotypic correlations in DA between arm and legs in a large dataset of human foetuses, which are not expected to emerge from mechanical loadings. Thus, the negative genetic correlations we show may explain the existence of negative phenotypic correlations without involving such a mechanical explanation. Moreover, the presence of negative genetic correlations constrains the evolution of higher or lower DA across traits. Finally, the fact that we found evidence for weak but significant environmental correlations between limbs may indicate a certain – albeit small – degree of developmental integration between bones of the front and hind limbs, which was not detected by phenotypic correlations.

### Concluding Remarks

Our results support that DA in long bones of rabbit limbs arises already during early development and that it is partially under genetic control. Assuming that symmetry is the target of selection of structures responsible for locomotion ([31]; but see [37], [38]), the existence of an asymmetric optimum seems unlikely. Indeed, Pelabon and Hansen [5] reported high coefficients of variation in DA in insect traits, arguing that even if an adaptive optimum for asymmetry existed it would be impossible to select for it given the high imprecision at which DA is expressed. Our results, with CVs ranging from 300 to 1000%, confirm their findings. Such imprecision together with the low level of DA (0.03–0.19%) in our dataset may suggest that the presence of DA is a by-product of the genetic architecture controlling trait development [5]. Alternatively, one could argue that DA arises during the fetal period as a pre-adaptation to behavioural lateralization in adults. Indeed, such a link between handedness and bone asymmetry has been established in humans [17], [39] and other primates [16], but so far not in other mammals [40]. Nevertheless, the fact that the bones in the forelimb consistently displayed higher levels of asymmetry than those in the hindlimbs might be explained by a stronger selection for symmetry in the lower extremities due to their more prominent role in locomotion. The existence of genetic correlations across bones of front and hind limbs would then limit the evolutionary potential of DA in the front limbs and thus the morphological adaptation to behavioural lateralisation.

Overall, our results do not preclude any of the above hypotheses to be confirmed. Instead they show possible future research directions in order to understand the basis of DA in locomotory traits, which remains an elusive, largely widespread trait. While foetal traits may offer an interesting model system to study the genetic architecture of asymmetry, it will also be crucial to study the changes in asymmetry during development and under different regimes of mechanic loading and exercise.
